# Association between hematoma expansion and short-term neurological outcomes in patients with spontaneous basal ganglia intracerebral hemorrhage at different blood pressure levels after blood pressure control: a single-center retrospective study

**DOI:** 10.3389/fneur.2026.1786695

**Published:** 2026-08-03

**Authors:** Hang Guo, Yehai Li

**Affiliations:** Department of Neurosurgery, Guangdong Second Provincial People's Hospital, Guangzhou, Guangdong, China

**Keywords:** blood pressure management, critical care, hematoma expansion, intracerebral hemorrhage, neurological outcomes

## Abstract

**Objective:**

To evaluate whether achieving a lower post-treatment systolic blood pressure (SBP) range during the acute phase was associated with reduced hematoma expansion and improved short-term neurological recovery in patients with spontaneous basal ganglia intracerebral hemorrhage (ICH).

**Methods:**

In this single-center retrospective study, 228 patients with spontaneous basal ganglia hemorrhage were analyzed after all had been managed under the same institutional early blood pressure (BP)-lowering protocol. For *post hoc* analysis, patients were retrospectively classified according to their achieved post-treatment SBP levels during the first 24 h into a lower achieved SBP group (120–140 mmHg) and a higher achieved SBP group (140–160 mmHg), rather than into prospectively assigned intensive vs. conventional treatment groups. Because group assignment reflected achieved BP levels rather than randomized treatment allocation, multivariable adjustment was performed to reduce confounding, although residual confounding and the limitations of complete-case analysis could not be fully excluded. The primary outcome was hematoma expansion (>6 ml or >33% increase on follow-up CT at 24–48 h). Secondary outcomes included absolute hematoma growth, neurological improvement (change in NIHSS score from admission to discharge), functional outcome at discharge (mRS), in-hospital mortality, hospital and ICU length of stay, and predefined safety endpoints.

**Results:**

The lower achieved SBP group had lower hematoma expansion rates (5.4% vs. 10.3%, *P* = 0.04) and less absolute hematoma growth [3.1 (IQR 1.2–5.0) ml vs. 9.4 (IQR 5.2–14.8) ml, *P* < 0.01]. The lower achieved SBP group demonstrated superior neurological recovery, with significantly greater NIHSS improvement (*P* < 0.05) and higher rates of favorable functional outcomes (mRS 0–2, *P* < 0.05). BP control was also more rapid and stable in the lower achieved SBP group, with reduced variability and longer maintenance within the target range. Multivariable analysis showed that belonging to the lower achieved SBP group was independently associated with a reduced risk of hematoma expansion (adjusted OR 0.52, 95% *CI* 0.30–0.90). Safety profiles were comparable, with no significant difference in serious adverse events.

**Conclusion:**

In this retrospective cohort, achieving a lower post-treatment SBP range during the first 24 h was associated with less hematoma expansion and better short-term neurological recovery in spontaneous basal ganglia hemorrhage.

## Introduction

Stroke is the second leading cause of death from non-communicable diseases worldwide (1). In 2021, intracerebral hemorrhage (ICH) accounted for approximately 28.8% of the estimated 11.9 million new strokes globally (2, 3). ICH is associated with substantial morbidity and mortality (4–6). Hypertension remains the most significant risk factor, with deep basal ganglia hemorrhage being the most common presentation (7, 8). Early hematoma expansion is a critical and modifiable determinant of prognosis (8).

Major clinical trials investigating early intensive blood pressure (BP) lowering have yielded nuanced and at times conflicting results (9–12). Current guidelines recommend rapid reduction of systolic blood pressure (SBP) to approximately 140 mmHg in acute ICH, as increased BP variability has been independently linked to poorer outcomes (9, 13–17). However, the evidence remains inconsistent, and most large-scale trials have not specifically focused on basal ganglia ICH. Moreover, relatively few studies have systematically examined how distinct patterns of BP control relate to hematoma evolution and early neurological recovery after ICH (18).

This study was designed to compare the associations of achieving a lower post-treatment SBP range (120–140 mmHg) vs. a higher range (140–160 mmHg) with hematoma expansion within 24–48 h in patients with spontaneous basal ganglia ICH. We also aimed to evaluate associations with neurological outcomes at hospital discharge and to explore the relationships between various SBP control parameters and short-term prognosis.

## Methods and materials

### Study design and patient population

**Figure 1:**
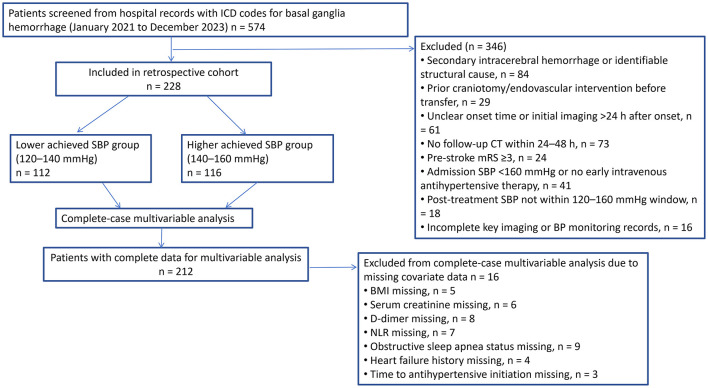
Flowchart of patient screening, retrospective achieved-SBP grouping, and complete-case analytic sample. A total of 574 patients with basal ganglia hemorrhage were screened, of whom 228 met the study eligibility criteria and were included in the retrospective cohort. All included patients were managed under the same institutional early blood pressure-lowering protocol and were subsequently categorized retrospectively according to the SBP levels actually achieved during the first 24 h after admission into the lower achieved SBP group (120–140 mmHg, *n* = 112) and the higher achieved SBP group (140–160 mmHg, *n* = 116). For multivariable analyses, complete-case analysis was performed, including 212 patients with complete covariate data; 16 patients were excluded from multivariable modeling because of missing data in at least one covariate.

This single-center retrospective cohort study was conducted from January 2021 to December 2023. All included patients were initially managed according to the same standardized institutional early BP-lowering protocol implemented in our stroke unit. For *post hoc* analysis, patients were retrospectively classified according to the SBP levels actually achieved during the first 24 h after admission rather than prospectively assigned treatment groups, introducing a potential risk of confounding by indication. During the study period, 574 potentially eligible patients with documented ICD codes for basal ganglia hemorrhage were screened. After application of the predefined inclusion and exclusion criteria, 228 patients were included in the final cohort (Figure 1).

### Patient selection criteria

**Inclusion Criteria:**

1. Age 18–80 years.
2. Spontaneous basal ganglia hemorrhage confirmed by non-contrast computed tomography (CT) scan.
3. Follow-up CT imaging completed within 24-48 h of admission for hematoma evaluation.
4. Admission SBP ≥160 mmHg with subsequent receipt of early intensive antihypertensive therapy.
5. Achievement of post-treatment SBP within 120–160 mmHg during hospitalization.
6. Complete medical records, including BP monitoring data and imaging documents.

**Exclusion Criteria:**

1. Secondary intracerebral hemorrhage due to identifiable causes.
2. Previous craniotomy or endovascular intervention performed at another institution prior to admission.
3. Unclear symptom onset time or initial imaging performed >24 h after symptom onset.
4. Significant pre-existing functional disability [pre-stroke modified Rankin Scale (mRS) score ≥3].
5. Missing critical imaging data or incomplete BP monitoring records precluding outcome assessment.

### Data collection and management

All patients underwent standardized assessment upon admission. Non-contrast head CT was performed immediately to confirm hemorrhage location and extent. Continuous BP monitoring was established using automated non-invasive BP devices. Clinical and demographic variables that could influence both BP control and clinical outcomes were collected using standardized electronic case report forms. These variables were grouped into demographic, medical history, neurological, temporal, radiological, and laboratory domains:

Demographic characteristics: age, sex, body mass index (BMI).

Medical history: hypertension, diabetes mellitus, chronic kidney disease, obesity, obstructive sleep apnea, coronary artery disease, heart failure, atrial fibrillation, smoking history, alcohol use, and use of anticoagulant or antiplatelet medications. These comorbidities were collected because they may influence vascular stiffness, volume status, sympathetic activation, antihypertensive responsiveness, and the likelihood of achieving lower SBP targets during the acute phase.

Neurological assessment: National Institutes of Health Stroke Scale (NIHSS) score and Glasgow Coma Scale (GCS) score on admission, to capture baseline neurological severity that might influence both the likelihood of achieving lower SBP and subsequent outcomes.

Time metrics: time from symptom onset to initial CT imaging and to initiation of antihypertensive therapy, as treatment timing may affect both achieved BP levels and hematoma progression.

Radiological parameters included baseline hematoma volume, calculated using the ABC/2 method, as well as the presence and severity of intraventricular extension. These imaging characteristics were collected because they may influence both hemodynamic control and clinical prognosis.

Laboratory parameters included complete blood count, coagulation profile, inflammatory markers, and renal function indices.

For data quality control, the research team conducted cross-validation of all extracted information against multiple hospital information systems, including the Electronic Medical Record (EMR), Picture Archiving and Communication System (PACS), and Nursing Information System. Discrepancies were resolved by consensus review by two independent investigators. All data were anonymized prior to analysis to ensure patient confidentiality.

### BP management protocol

#### Standardized early BP-lowering protocol

During the study period, all eligible patients were managed according to an institutional early intensive BP control protocol. Upon confirmation of basal ganglia hemorrhage on CT and documentation of elevated SBP, attending neurologists initiated intravenous antihypertensive therapy according to standardized medical orders.

The protocol specified:

Initial Management: For patients with admission SBP between 150 and 220 mmHg, intravenous antihypertensive therapy was initiated immediately after CT confirmation.

BP Targets: The institutional treatment goal was to reduce SBP into the 120–160 mmHg range within the first hour after admission, with emphasis on avoiding persistent SBP ≥160 mmHg and excessive reduction below 120 mmHg.

Maintenance Phase: Following initial reduction, SBP was maintained as consistently as possible within the 120–160 mmHg range for the subsequent 24 h.

Monitoring Frequency: Non-invasive BP measurements were obtained every 15 min during the first 2 h, every 30 min from 2 to 6 h, and at least hourly from 6 to 24 h.

Medication Administration: Continuous intravenous infusion of antihypertensive agents (primarily urapidil, nicardipine, or sodium nitroprusside) was administered via infusion pumps to ensure precise control and gradual BP reduction.

Dose Adjustment: Infusion rates were adjusted based on real-time BP measurements, with modifications made whenever two consecutive readings fell outside the target range.

Throughout the intensive management period, key clinical indicators including neurological status (NIHSS score changes), level of consciousness (GCS score), and renal function were closely monitored. Physicians aimed to balance aggressive BP reduction with maintenance of adequate cerebral perfusion.

#### Post-treatment BP level classification

For analytical purposes only, patients were retrospectively categorized according to the SBP levels they actually achieved during the first 24 h after hospital admission.

**Lower Achieved SBP Group (120–140 mmHg):** This group comprised patients whose SBP measurements were primarily sustained within the 120–140 mmHg range over the first day. The classification criteria required that: (a) the calculated mean SBP fell between 120 and 140 mmHg; (b) more than half of all individual SBP readings were within this same range; and (c) there was no documentation of sustained SBP levels at or above 160 mmHg.

**Higher Achieved SBP Group (140–160 mmHg):** Patients assigned to this group had SBP values that were mainly maintained within the 140–160 mmHg bracket during the same 24-h window. Assignment was based on: (a) a mean SBP within 140–160 mmHg, and (b) a majority (>50%) of individual measurements falling within this higher range.

The assignment to these groups was determined through a thorough review of all SBP readings documented from admission through the first 24 h, sourced from EMR and nursing charts. The research team computed several BP metrics, including the arithmetic mean SBP, a time-weighted average SBP, and the proportion of readings distributed across predefined tiers (< 120 mmHg, 120–140 mmHg, 140–160 mmHg, and ≥160 mmHg).

It is important to emphasize that this achieved-BP categorization was used for analysis only and did not represent prospective treatment allocation. Group membership neither guided bedside management nor reflected two predefined intervention strategies. Rather, this approach was used to examine whether differences in the SBP levels ultimately achieved after the same initial protocol were associated with subsequent hematoma evolution and short-term neurological outcomes. However, the achieved SBP level may also have reflected underlying patient characteristics, treatment responsiveness, vascular compliance, or initial disease severity, rather than BP exposure alone.

### Outcome measures

#### BP and hemodynamic assessment

Key parameters derived from SBP during the first 72 h included: (1) BP Level (mean SBP over first 24 h), (2) Speed of Control (time to first SBP < 140 mmHg), (3) Target Achievement (proportion of readings within 120–160 mmHg), and (4) BP Variability (quantified via standard deviation, coefficient of variation, average real variability, and max-min difference). These indices characterized the intensity, speed, and stability of BP control and served as primary exposure variables.

#### Imaging assessment

Hematoma volumes from initial and 24–48 h follow-up CT scans were measured using the ABC/2 method. Expansion was defined as an absolute increase >6 ml or relative growth >33%. Other metrics included absolute volume change, perilesional edema volume, midline shift (significant if ≥3 mm), and change in Graeb score. All imaging was independently assessed by two blinded clinicians; averaged results were used for analysis.

#### Neurological and short-term outcomes

The primary functional endpoint was the modified Rankin Scale (mRS) score at hospital discharge (favorable outcome = 0–2). Secondary measures included neurological improvement (ΔNIHSS, admission vs. discharge), hospital and ICU length of stay, and safety outcomes (hypotension, acute kidney injury, new cerebral infarction, pneumonia, deep vein thrombosis, and rebleeding).

The extent and pattern of missing data were reviewed before formal analysis. Because missingness was limited overall and did not affect the primary exposure classification or primary imaging outcome, complete-case analysis was used for multivariable modeling.

## Statistical analysis

Analyses were performed using SPSS 26.0 and R 4.3.1. Continuous variables were first assessed for distributional characteristics using visual inspection and normality testing. Variables with approximately normal distributions are presented as mean ± standard deviation and were compared using the independent-samples *t*-test, whereas skewed variables are presented as median (interquartile range) and were compared using the Mann–Whitney *U*-test. Categorical variables are presented as counts (percentages) and were compared using the χ^2^-test when expected cell counts were adequate; Fisher's exact test was used when expected counts were small. Baseline comparisons between the lower and higher achieved SBP groups included demographic variables, admission severity, radiological characteristics, laboratory indices, and comorbidities potentially related to difficulty in BP control, including diabetes mellitus, chronic kidney disease, obesity, obstructive sleep apnea, coronary artery disease, and heart failure.

Missing data were handled using a complete-case analysis. Among the final study cohort, 212 patients (93.0%) had complete data for all variables required for multivariable modeling, whereas 16 (7.0%) had missing data in at least one covariate and were therefore excluded from the complete-case multivariable analysis. Overall, missingness was limited and mainly involved laboratory and comorbidity-related variables rather than the primary exposure or primary outcome. Specifically, missing data were observed for body mass index in 5 patients (2.2%), serum creatinine in 6 patients (2.6%), D-dimer in 8 patients (3.5%), neutrophil-to-lymphocyte ratio in 7 patients (3.1%), obstructive sleep apnea status in 9 patients (3.9%), heart failure history in 4 patients (1.8%), and time to antihypertensive initiation in 3 patients (1.3%). No missing data were present for group classification, hematoma expansion status, admission NIHSS score, baseline hematoma volume, or intraventricular extension.

A complete-case approach was chosen because the proportion of missing data was relatively small, the study sample was moderate in size, and reliable auxiliary variables for multiple imputation were limited in this retrospective dataset. However, this approach may introduce bias if the excluded observations were not missing completely at random and may reduce statistical power by decreasing the effective sample size for multivariable analyses. To assess the robustness of the findings, a sensitivity analysis comparing baseline characteristics between complete cases and excluded incomplete cases was performed and did not show clinically meaningful differences in age, admission NIHSS score, baseline hematoma volume, or achieved SBP grouping.

To mitigate confounding arising from the retrospective classification by achieved SBP level, multivariable logistic regression was used to identify factors associated with hematoma expansion and favorable discharge mRS. Covariates were prespecified on the basis of clinical relevance, prior literature, and their potential association with both achieved BP levels and outcomes, rather than selected solely by univariable screening. These included age, sex, baseline NIHSS, baseline hematoma volume, intraventricular extension, BP variability indices, hospital length of stay, and major comorbidities related to BP control when available. These adjustments were intended to account, at least in part, for baseline differences in disease severity and clinical status that might influence both achieved BP levels and outcomes. Given the overall sample size of 228 patients, the multivariable models were designed to remain parsimonious; therefore, subgroup analyses should be interpreted as exploratory. A two-sided *P*-value < 0.05 was considered statistically significant. Residual confounding from unmeasured or incompletely measured factors could not be excluded.

## Results

### Baseline characteristics of the study population

The study cohort comprised 228 patients with spontaneous basal ganglia hemorrhage. Of these, 112 were classified into the lower achieved SBP group and 116 into the higher achieved SBP group according to the SBP levels actually achieved during the first 24 h after admission. As shown in Table 1, the two groups were broadly comparable with respect to age, sex, admission NIHSS score, GCS score, onset-to-imaging time, baseline hematoma volume, and intraventricular extension. However, comorbidities potentially related to difficulty in BP control tended to be more common in the higher achieved SBP group, including obesity (26.7% vs. 17.0%), diabetes mellitus (26.7% vs. 21.4%), chronic kidney disease (17.2% vs. 8.9%), obstructive sleep apnea (12.9% vs. 5.4%), coronary artery disease (19.8% vs. 16.1%), and heart failure (10.3% vs. 4.5%). This pattern suggests that failure to achieve lower SBP may, at least in part, reflect a greater burden of comorbidity affecting BP control rather than BP exposure alone.

**Table 1:** Baseline characteristics and blood pressure control-related comorbidities of patients in the lower and higher achieved SBP groups (*N* = 228).

Variable	Lower achieved SBP group (*n* = 112)	Higher achieved SBP group (*n* = 116)	*P*-value
Age, years (mean ±*SD*)	61.5 ± 10.1	62.1 ± 10.4	0.58
Male sex, *n* (%)	77 (68.8)	77 (66.4)	0.7
Body mass index, kg/m^2^ (mean ±*SD*)	25.6 ± 3.4	26.1 ± 3.8	0.29
Obesity, *n* (%)	19 (17.0)	31 (26.7)	0.07
History of hypertension, *n* (%)	86 (76.8)	78 (67.2)	0.1
Diabetes mellitus, *n* (%)	24 (21.4)	31 (26.7)	0.35
Chronic kidney disease, *n* (%)	10 (8.9)	20 (17.2)	0.06
Obstructive sleep apnea, *n* (%)	6 (5.4)	15 (12.9)	0.05
Coronary artery disease, *n* (%)	18 (16.1)	23 (19.8)	0.47
Heart failure, *n* (%)	5 (4.5)	12 (10.3)	0.09
Atrial fibrillation, *n* (%)	11 (9.8)	14 (12.1)	0.59
Anticoagulant/antiplatelet use, *n* (%)	14 (12.5)	12 (10.3)	0.6
Smoking history, *n* (%)	36 (32.1)	34 (29.3)	0.65
Alcohol use, *n* (%)	30 (26.8)	28 (24.1)	0.63
Admission NIHSS score, median (IQR)	12 (8–18)	13 (8–17)	0.72
Admission GCS score, median (IQR)	11 (9–14)	11 (9–14)	0.88
Onset-to-imaging time, h (mean ±*SD*)	4.1 ± 2.3	4.3 ± 2.5	0.54
Time to antihypertensive initiation, h (mean ±*SD*)	1.6 ± 0.8	1.8 ± 0.9	0.11
Baseline hematoma volume, ml, median (IQR)	18.6 (12.2–28.4)	19.1 (12.0–29.7)	0.81
Intraventricular extension, *n* (%)	28 (25.0)	30 (25.9)	0.87
Graeb score, median (IQR)	2 (0–4)	2 (0–4)	0.92
INR (mean ±*SD*)	1.03 ± 0.12	1.04 ± 0.11	0.46
D-dimer, mg/L, median (IQR)	0.78 (0.42–1.25)	0.81 (0.45–1.31)	0.67
NLR, median (IQR)	4.9 (3.1–7.3)	5.1 (3.2–7.5)	0.59
Serum creatinine, μmol/L (mean ± SD)	78.4 ± 16.7	84.9 ± 21.3	0.03
Serum glucose, mmol/L (mean ± SD)	7.8 ± 2.1	8.2 ± 2.4	0.18

Values are presented as mean ± standard deviation (SD), median (interquartile range [IQR]), or number (percentage), as appropriate. *P*-values were calculated using the independent-samples *t*-test, Mann–Whitney *U*-test, χ^2^-test, or Fisher's exact test, as appropriate. NIHSS, National Institutes of Health Stroke Scale; GCS, Glasgow coma scale; INR, international normalized ratio; NLR, neutrophil-to-lymphocyte ratio; SBP, systolic blood pressure.

### Hemodynamic patterns

Patients in the lower achieved SBP group exhibited a more rapid and pronounced reduction in BP. As shown in Table 2, the lower achieved SBP group demonstrated more rapid and sustained BP control than the higher achieved SBP group. Mean SBP was consistently lower across all early time windows, time to first SBP < 140 mmHg was shorter, and the proportion of readings within the target range during the first 24 h was higher. In addition, BP variability was significantly lower in the lower achieved SBP group, and exposure to extreme SBP elevations (including readings >180 mmHg and sustained SBP ≥160 mmHg) was markedly less frequent. Furthermore, all indices of BP variability were markedly reduced in the lower achieved SBP group, including SBP SD, coefficient of variation, average real variability, and max–min SBP difference.

**Table 2:** Blood pressure control and hemodynamic profile in the lower and higher achieved SBP groups within 72 h after admission.

Variable	Lower achieved SBP group (*n* = 112)	Higher achieved SBP group (*n* = 116)	*P*-value
Time-stratified mean SBP			
Mean SBP 0–1 h, mmHg (mean ±*SD*)	150 ± 18	165 ± 20	< 0.001
Mean SBP 1–6 h, mmHg (mean ±*SD*)	142 ± 16	158 ± 19	< 0.001
Mean SBP 6–24 h, mmHg (mean ±*SD*)	138 ± 14	152 ± 18	< 0.001
Mean SBP 24–72 h, mmHg (mean ±*SD*)	136 ± 15	145 ± 16	0.01
Blood pressure target achievement			
Time to first SBP < 140 mmHg, h, median (IQR)	0.8 (0.5–1.4)	3.2 (2.1–5.6)	< 0.001
Proportion of readings within target range (120–160 mmHg) during first 24 h, %	78	46	< 0.001
Time maintained within target range during first 24 h, h (mean ±*SD*)	18.7 ± 4.3	11.2 ± 5.1	< 0.001
Blood pressure variability metrics during first 24 h			
SBP SD, mmHg (mean ±*SD*)	11.2 ± 4.3	16.5 ± 5.1	< 0.001
SBP coefficient of variation (mean ±*SD*)	0.081 ± 0.02	0.110 ± 0.03	< 0.001
SBP average real variability, mmHg (mean ±*SD*)	8.4 ± 3.1	12.6 ± 4.2	< 0.001
Max–min SBP difference, mmHg (mean ±*SD*)	38 ± 10	52 ± 13	< 0.001
Distribution of SBP readings during first 24 h			
Proportion of readings < 120 mmHg, %	8	3	0.04
Proportion of readings 120–140 mmHg, %	54	21	< 0.001
Proportion of readings 140–160 mmHg, %	24	49	< 0.001
Proportion of readings ≥160 mmHg, %	14	27	< 0.001
Extreme blood pressure exposure			
Proportion of readings >180 mmHg, %	4	12	< 0.001
Patients with any SBP reading >180 mmHg, *n* (%)	18 (16.1)	41 (35.3)	0.001
Patients with sustained SBP ≥160 mmHg for >2 h, *n* (%)	12 (10.7)	37 (31.9)	< 0.001

SBP, systolic blood pressure; SD, standard deviation; IQR, interquartile range. Values are presented as mean ± *SD*, median (IQR), number (percentage), or percentage, as appropriate. *P*-values were calculated using the independent-samples *t*-test, Mann–Whitney *U*-test, χ^2^-test, or Fisher's exact test, as appropriate.

### Safety profile and in-hospital complications

As shown in Table 3, the overall incidence of in-hospital safety events and complications was comparable between groups. Hypotension events were numerically more frequent in the lower achieved SBP group (12.5% vs. 6.9%, *P* = 0.12), whereas rebleeding (5.4% vs. 10.3%, *P* = 0.12), pneumonia (16.1% vs. 24.1%, *P* = 0.12), neurological worsening (6.3% vs. 12.9%, *P* = 0.09), and in-hospital mortality (4.5% vs. 7.8%, *P* = 0.28) tended to be less frequent. No significant between-group differences were observed for acute kidney injury, thromboembolic events, ICU admission, or mechanical ventilation.

**Table 3:** In-hospital safety outcomes and complications in the lower and higher achieved SBP groups.

Variable	Lower achieved SBP group (*n* = 112)	Higher achieved SBP group (*n* = 116)	*P*-value
Hemorrhagic and ischemic events			
Rebleeding, *n* (%)	6 (5.4)	12 (10.3)	0.12
New-onset intracerebral hemorrhage elsewhere, *n* (%)	2 (1.8)	4 (3.4)	0.42
New-onset ischemic stroke, *n* (%)	3 (2.7)	7 (6.0)	0.2
Blood pressure-related adverse events			
Hypotension events, *n* (%)	14 (12.5)	8 (6.9)	0.12
Severe hypotension (SBP < 90 mmHg), *n* (%)	3 (2.7)	2 (1.7)	0.67
Renal and metabolic complications			
Acute kidney injury (KDIGO-defined), *n* (%)	8 (7.1)	10 (8.6)	0.68
Need for renal replacement therapy, *n* (%)	1 (0.9)	2 (1.7)	0.61
Hypernatremia (>155 mmol/L), *n* (%)	5 (4.5)	6 (5.2)	0.82
Hypoglycemia (< 3.5 mmol/L), *n* (%)	3 (2.7)	5 (4.3)	0.49
Infectious complications			
Pneumonia, *n* (%)	18 (16.1)	28 (24.1)	0.12
Urinary tract infection, *n* (%)	10 (8.9)	14 (12.1)	0.42
Sepsis, *n* (%)	4 (3.6)	9 (7.8)	0.17
Thromboembolic events			
Deep vein thrombosis, *n* (%)	5 (4.5)	9 (7.8)	0.28
Pulmonary embolism, *n* (%)	1 (0.9)	3 (2.6)	0.29
Neurological deterioration metrics			
Neurological worsening (>4-point NIHSS increase), *n* (%)	7 (6.3)	15 (12.9)	0.09
New-onset seizures, *n* (%)	4 (3.6)	8 (6.9)	0.23
Resource utilization and mortality			
ICU admission, *n* (%)	44 (39.3)	52 (44.8)	0.4
ICU length of stay, days, median (IQR)	3.2 (2.0–5.0)	4.1 (3.0–7.0)	0.08
Need for mechanical ventilation, *n* (%)	12 (10.7)	18 (15.5)	0.29
In-hospital mortality, *n* (%)	5 (4.5)	9 (7.8)	0.28

Values are presented as number (percentage) or median (interquartile range [IQR]), as appropriate. *P*-values were calculated using the χ^2^-test, Fisher's exact test, or Mann–Whitney *U*-test, as appropriate. SBP, systolic blood pressure; NIHSS, National Institutes of Health Stroke Scale; KDIGO, kidney disease: improving global outcomes; ICU, intensive care unit.

### Hematoma progression and imaging findings

**Figure 2:**
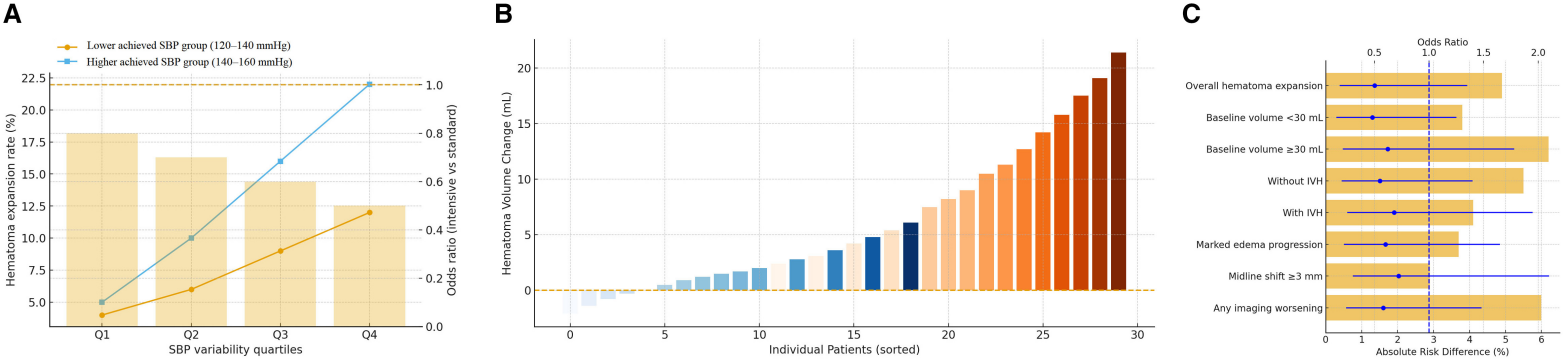
Radiological progression and hematoma expansion. **(A)** Combined bar-and-point plot of predefined radiological outcomes comparing the lower achieved SBP group and the higher achieved SBP group. The yellow horizontal bars represent the absolute risk difference (%) for each imaging outcome between groups, referenced to the lower *x*-axis. The blue points and horizontal blue lines represent the corresponding odds ratios and their 95% confidence intervals, referenced to the upper *x*-axis. The dashed vertical blue line indicates the null value for the odds ratio (OR = 1.0). **(B)** Waterfall plot of individual changes in hematoma volume, sorted from smallest to largest value. Each bar represents one patient, and bar color reflects the magnitude of hematoma volume change, with cooler colors indicating smaller or negative changes and warmer colors indicating larger increases. The dashed horizontal line at 0 ml indicates no change in hematoma volume. **(C)** Combined bar-and-line plot showing hematoma expansion across quartiles of systolic blood pressure variability. The yellow bars represent the overall hematoma expansion rate (%) in each SBP variability quartile, referenced to the left *y*-axis. The orange circular line and the blue square line represent the odds ratios for hematoma expansion in the lower achieved SBP group and the higher achieved SBP group, respectively, referenced to the right *y*-axis. The dashed horizontal line on the right axis indicates the null value (OR = 1.0).

Belonging to the lower achieved SBP group was associated with a significantly lower incidence of hematoma expansion (*P* = 0.04). This group also demonstrated less absolute growth in hematoma volume (*P* < 0.01) and a more modest increase in peri-hematomal edema (*P* < 0.01), as presented in Table 4. The trend toward reduced risk was evident across related imaging endpoints (Figure 2A). An analysis of individual patient data revealed less hematoma growth in the lower achieved SBP group (Figure 2B). Stratified analyses indicated that the risk of expansion increased with greater BP variability in both groups, but this relationship was attenuated in the lower achieved SBP group (Figure 2C).

**Table 4:** Imaging outcomes and hematoma progression within 24–48 h in the lower and higher achieved SBP groups.

Variable	Lower achieved SBP group (*n* = 112)	Higher achieved SBP group (*n* = 116)	*P*-value
Hematoma expansion, *n* (%)	6 (5.4%)	12 (10.3%)	0.04
Hematoma expansion volume, ml, median (IQR)	3.1 (1.2–5.0)	9.4 (5.2–14.8)	< 0.01
Patients with expansion >10 ml, *n* (%)	8 (7.1%)	22 (19.0%)	< 0.01
Perihematomal edema increase, ml (mean ±*SD*)	4.6 ± 2.1	7.8 ± 3.4	< 0.01
Midline shift progression ≥3 mm, *n* (%)	7 (6.3%)	15 (12.9%)	0.09
Graeb score change, median (IQR)	0 (0–1)	1 (0–2)	0.03
New intraventricular extension, *n* (%)	3 (2.7%)	7 (6.0%)	0.2
New-onset infarction, *n* (%)	3 (2.7%)	7 (6.0%)	0.2
Rebleeding, *n* (%)	5 (4.5%)	12 (10.3%)	0.08
Any imaging worsening, *n* (%)	14 (12.5%)	29 (25.0%)	0.02

Hematoma expansion was defined as an absolute increase of >6 ml or a relative increase of >33% on follow-up CT. Rebleeding refers to newly detected bleeding within or adjacent to the original hematoma cavity and may occur independently of the quantitative hematoma expansion definition.

### Neurological improvement and short-term clinical outcomes

**Figure 3:**
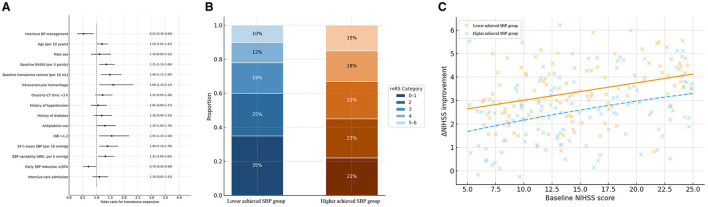
Neurological changes and short-term outcomes. **(A)** Box plots of NIHSS scores at admission and discharge for the lower achieved SBP group and the higher achieved SBP group, showing the distribution of neurological scores at the two time points. **(B)** Stacked bar chart showing the distribution of mRS scores at discharge, illustrating the proportional composition of different mRS grades in the lower achieved SBP group and the higher achieved SBP group. **(C)** Scatter plot with baseline NIHSS score on the *x*-axis and ΔNIHSS on the *y*-axis, displaying data points and fitted trend lines for the lower achieved SBP group and the higher achieved SBP group to illustrate the distribution and trend of neurological improvement across different baseline severity levels.

Patients in the lower achieved SBP group demonstrated superior neurological recovery. They had lower NIHSS scores at discharge (*P* < 0.01), a greater degree of improvement in NIHSS from admission (ΔNIHSS: 7.1 ± 3.2 vs. 5.0 ± 3.0, *P* < 0.01), and a higher proportion achieving a favorable functional outcome (*P* = 0.02), as shown in Table 5. The trajectory of mean NIHSS scores over time is depicted in Figure 3A. The distribution of mRS categories at discharge and functional outcomes stratified by baseline NIHSS are illustrated in Figures 3B, C, respectively. Additionally, the median hospital length of stay was shorter in the lower achieved SBP group (*P* = 0.03).

**Table 5:** Neurological recovery and short-term clinical outcomes in the lower and higher achieved SBP groups.

Outcome	Lower achieved SBP group (*n* = 112)	Higher achieved SBP group (*n* = 116)	*P*-value
NIHSS at admission, mean ±*SD*	13.2 ± 4.1	13.0 ± 4.3	0.72
NIHSS at discharge, mean ±*SD*	6.1 ± 3.5	8.0 ± 3.9	< 0.01
ΔNIHSS (admission – discharge), mean ±*SD*	7.1 ± 3.2	5.0 ± 3.0	< 0.01
Patients with ΔNIHSS ≥ 4, *n* (%)	78 (69.6%)	61 (52.6%)	0.01
Patients with ΔNIHSS ≥ 8, *n* (%)	34 (30.4%)	18 (15.5%)	0.01
Favorable outcome (mRS 0–2), *n* (%)	62 (55.4%)	46 (39.7%)	0.02
mRS 0–1 at discharge, *n* (%)	39 (34.8%)	26 (22.4%)	0.04
Poor outcome (mRS 4–6), *n* (%)	25 (22.3%)	38 (32.8%)	0.08
In-hospital mortality, *n* (%)	5 (4.5%)	9 (7.8%)	0.28
Length of hospital stay, days, median (IQR)	12 (9–16)	14 (10–18)	0.03
ICU stay, days, median (IQR)	3 (2–5)	4 (3–7)	0.04

Values are presented as mean ± standard deviation (SD), number (percentage), or median [interquartile range (IQR)], as appropriate. Comparisons between groups were performed using the independent-samples *t*-test, Mann–Whitney *U*-test, χ^2^-test, or Fisher's exact test, as appropriate. NIHSS, National Institutes of Health Stroke Scale; mRS, modified Rankin scale; ICU, intensive care unit; SBP, systolic blood pressure.

### Missing data summary

Missing data were limited overall. Of the 228 included patients, 212 (93.0%) had complete data for all variables required in the multivariable models, whereas 16 patients (7.0%) were excluded from complete-case multivariable analyses because of missing data in at least one covariate. The variables with missing values were body mass index (5/228, 2.2%), serum creatinine (6/228, 2.6%), D-dimer (8/228, 3.5%), neutrophil-to-lymphocyte ratio (7/228, 3.1%), obstructive sleep apnea status (9/228, 3.9%), heart failure history (4/228, 1.8%), and time to antihypertensive initiation (3/228, 1.3%). No missing data were observed for achieved SBP grouping, hematoma expansion, baseline NIHSS, baseline hematoma volume, or intraventricular extension.

### Multivariable and subgroup analyses

**Figure 4:**
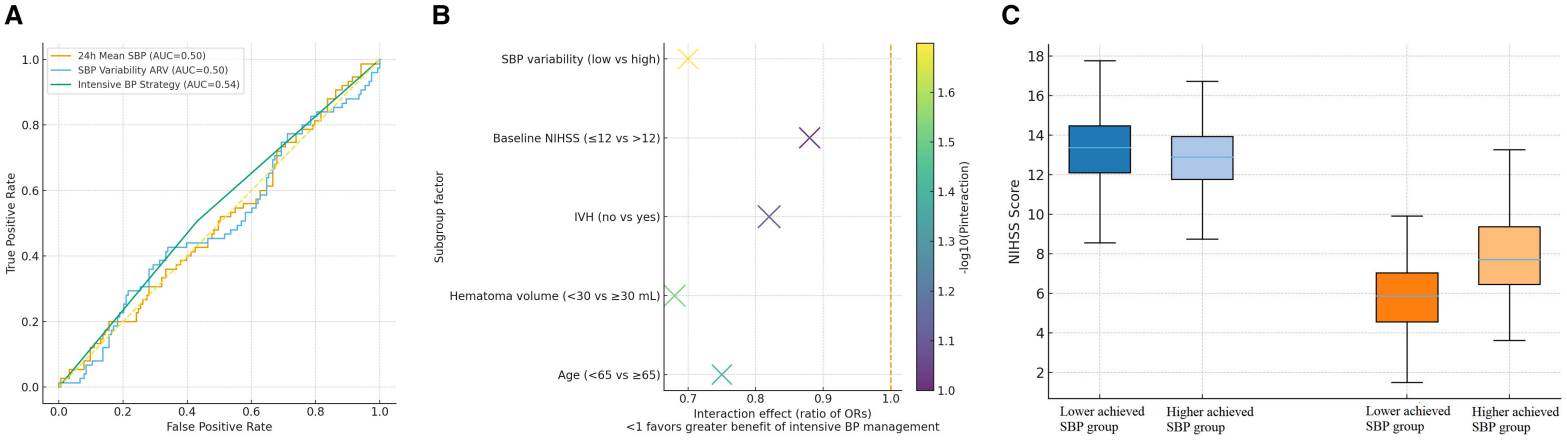
Results of multivariable regression and subgroup analyses. **(A)** Forest plot from the multivariable logistic regression analysis, displaying the odds ratios and their 95% confidence intervals for the association between various covariates and the occurrence of hematoma expansion. The dashed line indicates the line of null effect, showing the direction and magnitude of association for each factor. **(B)** Bubble plot illustrating subgroup interaction effects, showing the ratio of odds ratios for the association between lower achieved SBP and hematoma expansion across different subgroup factors. **(C)** Receiver operating characteristic (ROC) curves comparing the discriminatory ability of mean systolic blood pressure over the first 24 h, the systolic blood pressure variability index, and achieved SBP grouping for predicting hematoma expansion.

In the complete-case multivariable analysis including 212 patients, classification into the lower achieved SBP group remained independently associated with a reduced risk of hematoma expansion [adjusted odds ratio (aOR) 0.52, 95% confidence interval (CI) 0.30–0.90, *P* = 0.02]. Nevertheless, because the grouping was based on achieved rather than assigned BP levels, this association should not be interpreted as definitive evidence of causality. A lower mean SBP and reduced BP variability were also identified as independent protective factors (Figure 4A). Subgroup analyses suggested that the association between lower achieved SBP and reduced hematoma expansion was more pronounced in patients younger than 65 years, those with a baseline hematoma volume < 30 ml, and those with lower BP variability (*P* for interaction < 0.05 for each subgroup; Figure 4B). However, BP parameters alone demonstrated only limited predictive accuracy for hematoma expansion, with an area under the receiver operating characteristic curve (AUC) of approximately 0.6 (Figure 4C). A sensitivity comparison between complete cases and excluded incomplete cases did not show clinically meaningful differences in key baseline variables, supporting the stability of the complete-case analytic sample.

## Discussion

This retrospective single-center analysis investigated the association between post-treatment SBP levels and clinical outcomes in patients with spontaneous basal ganglia intracerebral hemorrhage. All enrolled patients initially received the same institutional BP-lowering protocol, after which they were retrospectively stratified for *post hoc* analysis according to the SBP range they actually achieved during the first 24 h, rather than according to prospectively assigned treatment intensity. Accordingly, the observed between-group differences should be interpreted in light of possible confounding by indication, because patients who achieved lower SBP may also have differed systematically in baseline physiology, treatment responsiveness, or disease severity. Expert guidelines advocate for reducing SBP to around 140 mmHg in the acute setting to mitigate secondary brain injury (19).

Our findings indicate that patients who achieved and maintained lower post-treatment SBP levels (120–140 mmHg) had less hematoma expansion and more favorable short-term functional status at discharge than those who remained in the higher achieved SBP range (140–160 mmHg), without a concomitant rise in major in-hospital complications. However, these differences may not be attributable solely to BP level itself. In our cohort, patients in the higher achieved SBP group tended to have a greater burden of comorbidities that may impair BP control, including chronic kidney disease, obesity, obstructive sleep apnea, coronary artery disease, and heart failure. These conditions may contribute to increased arterial stiffness, altered intravascular volume regulation, sympathetic overactivity, reduced responsiveness to intravenous antihypertensive agents, or greater hemodynamic instability, thereby making it more difficult to achieve lower SBP targets in the acute phase. Our observations are in agreement with a secondary analysis of the ATACH-2 trial, which reported that intensive BP lowering substantially curtailed hematoma growth in patients with deep intracerebral hemorrhage (11). Additional support comes from a study noting that early intensive lowering reduced expansion rates specifically in patients with active bleeding (17), and a meta-analysis confirming that rapid BP control achieved with nicardipine lowered both the risk of expansion and 90-day disability (20). It should be noted, however, that a pooled analysis of the INTERACT trials did not demonstrate a significant overall effect on hematoma volume (21). Several factors may explain why our findings appear more favorable. First, our study focused specifically on spontaneous basal ganglia ICH, whereas large multicenter trials typically enrolled more heterogeneous ICH populations with broader anatomical and clinical variation. Second, our exposure groups were defined according to SBP levels actually achieved after treatment rather than prospectively assigned BP targets, which may enrich the lower achieved SBP group for patients with more favorable vascular responsiveness or lower physiological instability. Third, our study assessed early radiological progression and short-term in-hospital outcomes, whereas major randomized trials often prioritized longer-term endpoints such as 90-day functional outcome, which may be influenced by many post-acute factors beyond early BP control. Fourth, differences in treatment timing, antihypertensive titration, baseline hematoma severity, and center-level practice patterns may also have contributed to the discrepancy. Taken together, our data suggest that lower achieved SBP levels may be linked to attenuation of the ongoing hemorrhagic process in basal ganglia hemorrhage; however, unlike randomized comparisons, our observational design cannot fully separate BP effects from patient-level factors associated with treatment response. The unequal distribution of these comorbidities is also important for interpretation of the outcome data. Patients with chronic kidney disease, diabetes, obesity, coronary artery disease, or heart failure may have had both greater difficulty achieving lower SBP and a higher baseline risk of adverse neurological or systemic outcomes. Therefore, the observed differences between achieved-SBP groups may reflect, at least in part, the combined effects of BP exposure and underlying comorbidity burden. This reinforces the need to interpret the associations in this study cautiously and not as evidence that achieved SBP alone fully explains the between-group outcome differences.

Furthermore, patients in our intensive BP-lowering cohort showed more pronounced short-term neurological recovery. This is congruent with a recent meta-analysis which found that intensive lowering elevated the proportion of patients achieving an excellent functional outcome (mRS 0–2) at 3 months (22). Other reports have similarly described increased odds of functional independence with rapid BP reduction (17), and pooled INTERACT data revealed benefits in mortality reduction and long-term recovery, particularly when intervention commenced within 3 h of onset (23, 24). In contrast, the ATACH-2 trial did not find a significant improvement in 90-day outcomes within its basal ganglia subgroup (25). This discrepancy may reflect several important design differences. ATACH-2 evaluated an assigned intensive BP-lowering strategy within a randomized framework, whereas our study compared retrospectively defined achieved-SBP groups after a shared treatment protocol. As a result, our lower achieved SBP group may have included patients who were inherently more likely to respond to treatment and to experience more favorable early recovery. In addition, ATACH-2 emphasized longer-term functional outcome, whereas our study focused on short-term in-hospital neurological recovery and discharge status. Our single-center cohort may also have differed from ATACH-2 in baseline severity distribution, local BP titration practice, case selection, and supportive care patterns. These differences limit direct comparability and suggest that our findings may be more reflective of early in-hospital associations in a selected basal ganglia ICH population than of a generalizable causal treatment effect. On balance, our study supports an association between lower achieved post-treatment SBP and better early neurological recovery, while recognizing that the retrospective achieved-BP grouping design does not allow firm causal inference regarding the neuroprotective effect of BP lowering itself (26).

Safety evaluations showed that our intensive protocol was not associated with a significant increase in major adverse events. Likewise, magnetic resonance imaging studies have reported no elevated risk of new ischemic lesions following aggressive BP reduction (27), and moderate lowering is generally not expected to cause cerebral hypoperfusion. Notably, in patients presenting with extremely high initial SBP (≥220 mmHg), intensive lowering failed to improve outcomes and was linked to an increased risk of acute kidney injury (25)—a complication not observed in our patient population, a finding consistent with other meta-analyses (22). Some evidence even suggests that intensive therapy may reduce the incidence of serious adverse events (21). Overall, a strategy of moderately intensive BP control appears to be generally safe, potentially reducing secondary neurological damage without introducing major systemic risks, although close hemodynamic monitoring remains essential to prevent organ hypoperfusion.

Our study highlights opportunities to refine BP management based on individual patient profiles.

Because our analysis was based on retrospectively defined groups according to achieved BP levels after a shared initial protocol, rather than prospectively assigned treatment regimens, the findings should be interpreted as observational associations that may inform, but not establish, optimal BP targets for individualized titration. Although we adjusted for several measured confounders, including age, sex, baseline NIHSS, baseline hematoma volume, intraventricular extension, BP variability, hospital length of stay, and major comorbidities related to BP control when available, residual confounding from unmeasured factors—such as vascular compliance, differential medication responsiveness, premorbid BP characteristics, and other clinical determinants of BP control—remains possible. In addition, because multivariable modeling was based on complete-case analysis, some loss of statistical efficiency was unavoidable, and bias may have been introduced if missing covariate data were not completely random. The baseline distribution of CKD, obesity, obstructive sleep apnea, coronary artery disease, and heart failure in the higher achieved SBP group further supports the likelihood that difficulty in BP control was influenced by patient-level clinical factors. In clinical practice, management strategies should account for the patient's premorbid BP and cerebral perfusion status (27). Intensive lowering may promote the restoration of cerebral hemodynamic balance by attenuating transmural pressure gradients, lessening compression around the hematoma, and reducing mechanical stress on compromised vessels (21). Placing our results alongside those from international studies suggests that an “early, rapid, and sustained” BP-lowering approach may be beneficial in selected patients with basal ganglia intracerebral hemorrhage (28); however, the single-center retrospective design, achieved-BP grouping strategy, and short-term endpoint focus of our study limit direct external generalizability to broader ICH populations.

## Limitations

Several limitations must be acknowledged. First, the single-center design and modest sample size may limit generalizability. Second, as a retrospective study, unmeasured confounders and temporal trends in care quality could influence outcomes. In particular, the exposure groups were defined retrospectively according to achieved SBP levels rather than prospectively assigned interventions, which increases the risk of confounding by indication and limits causal inference. Patients who achieved lower SBP may have differed from those with higher achieved SBP in ways not fully captured in our dataset, including vascular compliance, antihypertensive responsiveness, premorbid BP profile, autonomic status, and other markers of clinical stability. Although multivariable adjustment was performed, such residual confounding cannot be completely eliminated. Third, although the amount of missing data was relatively small, the multivariable analyses relied on complete-case analysis, which excluded patients with missing covariate data. If those missing observations were not missing completely at random, selection bias may have been introduced, and statistical power may have been modestly reduced. Fourth, the follow-up was short-term; long-term prognostic impact remains unclear. Additionally, we could not stratify by individualized optimal BP targets, and the lack of cerebral perfusion data precluded direct mechanistic assessment. Future research should validate these findings in larger, multicenter prospective cohorts, consider multiple imputation when appropriate auxiliary variables are available, and employ dynamic perfusion monitoring and advanced imaging to refine personalized BP strategies. Exploring combinations of intensive BP lowering with other neuroprotective therapies could further improve ICH outcomes.

## Conclusion

In this retrospective cohort of patients with spontaneous basal ganglia ICH, achieving lower post-treatment SBP levels (120–140 mmHg) during the first 24 h was associated with reduced hematoma expansion and better short-term neurological function at discharge compared with achieving higher SBP levels (140–160 mmHg), without a significant increase in overall in-hospital complications. These findings suggest that achieving and maintaining lower SBP levels may be associated with less hematoma progression and better early outcomes; however, given the potential for confounding by indication in this retrospective analysis, confirmation in larger prospective studies is required before inferring a causal benefit or refining optimal post-treatment BP targets.

## Data Availability

The raw data supporting the conclusions of this article will be made available by the authors, without undue reservation.
